# Longitudinal time-lapse in vivo micro-CT reveals differential patterns of peri-implant bone changes after subclinical bacterial infection in a rat model

**DOI:** 10.1038/s41598-020-77770-z

**Published:** 2020-12-01

**Authors:** Vincent A. Stadelmann, Keith Thompson, Stephan Zeiter, Karin Camenisch, Ursula Styger, Sheila Patrick, Andrew McDowell, Dirk Nehrbass, R. Geoff Richards, T. Fintan Moriarty

**Affiliations:** 1 AO Research Institute Davos, Clavadelerstrasse 8, 7270 Davos Platz, Switzerland; 2 Schulthess Klinik, Department of Research and Development, Lengghalde 2, 8008 Zurich, Switzerland; 3 The Wellcome-Wolfson Institute for Experimental Medicine, Queen’s University Belfast, Lisburn Rd, Belfast, BT9 7AE UK; 4 Nutrition Innovation Centre for Food and Health (NICHE), School of Biomedical Sciences, Ulster University, Cromore Road, Coleraine, BT52 1SA UK

**Keywords:** Biological techniques, Microbiology, Biomarkers, Medical research, Pathogenesis

## Abstract

Subclinical infection associated with orthopedic devices can be challenging to diagnose. The goal of this study was to evaluate longitudinal, microcomputed tomography (microCT) imaging in a rat model of subclinical orthopedic device-related infection caused by *Staphylococcus epidermidis* and four different *Cutibacterium* (previously *Propionibacterium*) *acnes* strains, and compare outcomes with non-inoculated and historical *S. aureus-*inoculated controls. Sterile screws or screws colonized with bacteria were placed in the tibia of 38 adult Wistar rats [n = 6 sterile screws; n = 6 *S. epidermidis-*colonized screws; n = 26 *C. acnes-*colonized screws (covering all three main subspecies)]. Regular microCT scans were taken over 28 days and processed for quantitative time-lapse imaging with dynamic histomorphometry. At euthanasia, tissues were processed for semiquantitative histopathology or quantitative bacteriology. All rats receiving sterile screws were culture-negative at euthanasia and displayed progressive bony encapsulation of the screw. All rats inoculated with *S. epidermidis*-colonized screws were culture-positive and displayed minor changes in peri-implant bone, characteristic of subclinical infection. Five of the 17 rats in the *C. acnes* inoculated group were culture positive at euthanasia and displayed bone changes at the interface of the screw and bone, but not deeper in the peri-implant bone. Dynamic histomorphometry revealed significant differences in osseointegration, bone remodeling and periosteal reactions between groups that were not measurable by visual observation of still microCT images. Our study illustrates the added value of merging 3D microCT data from subsequent timepoints and producing inherently richer 4D data for the detection and characterization of subclinical orthopedic infections, whilst also reducing animal use.

## Introduction

Complications associated with orthopedic implants can result in pain, delayed healing and restricted function of the affected limb. Some of the more common complications include orthopedic device-related infection (ODRI) and aseptic loosening. *Staphylococcus aureus* is the predominant bacterial species responsible for acute ODRIs, with a prevalence of up to 40%^1^. However, a significant portion of ODRIs are subclinical or delayed infections, and are often caused by less virulent pathogens, such as *Staphylococcus epidermidis*^2,3^ or *Cutibacterium* (previously *Propionibacterium*) *acnes*^4^. In particular, *C. acnes* has been reported to cause up to 56% of infections related to shoulder prostheses^5^ where clinical symptoms are often subtle, such as slight tenderness of the affected region with a lack of swelling and mild, if any, osteolysis.

The early detection of complications such as peri-implant osteolysis could improve patient outcome by facilitating early intervention strategies and potentially avoid the need for implant removal. Conventional radiographs are not, however, sufficiently sensitive to detect the subtle changes in peri-implant bone induced by ODRI at the early stages of an infection, or those caused by low-virulence pathogens. In contrast, computed tomography (CT) can show bone structures with high precision and would have the potential to detect these changes. In laboratory animals, microcomputed tomography (microCT) has been widely adopted to assess structural bone changes in vivo^6,7^. Furthermore, by combining microCT images from successive timepoints, known as time-lapse imaging, it is possible to monitor the patterns of changes by computing dynamic histomorphometric indices, such as bone formation and resorption rates in a noninvasive manner^8,9^. Recently, time-lapse in vivo microCT was successfully applied by Kettenberger et al*.* to quantify the impact of bisphosphonates on periprosthetic bone changes^10^ and by our group to quantify the pronounced peri-implant bone changes following acute *S. aureus*-induced ODRI in a rat model^11^.

In the present study, we apply time-lapse in vivo microCT with revised image processing algorithms to characterize the progression of ODRI induced by the low-virulence pathogens *S. epidermidis* and *C. acnes* using the same rat model. Our aim was to determine whether in vivo microCT has sufficient sensitivity to identify differences in peri-implant bone changes in the setting of subclinical infections.

## Materials and methods

### Ethics statement

Ethical approval to perform this animal study and experimental protocols were granted by the ethical committee of the canton of Graubünden in Switzerland (approval number: 2012_21) to ensure the ethical care and use of laboratory animals in experimental research. All animal studies were carried out in the AAALAC (Association for Assessment and Accreditation of Laboratory Animal Care) accredited Research Institute. The animal model is adapted from a model that has previously been established at the AO Research Institute Davos, Switzerland and was used to monitor peri-implant bone changes around screws implanted in the tibia of young, old and osteoporotic rats^12^. Also, the AO Institutional Review Board reviewed and approved this study (approval number: AR2010_05). All methods were performed in accordance with ISO 9001:2015, Good Laboratory Practice (GLP), and AAALAC relevant regulations.

### Study outline

In this study a total of 38 animals were used. The implanted screws were either sterile (n = 6) or colonized with *S. epidermidis* (n = 6) or *C. acnes* (n = 26); the *C. acnes* group comprised strains representing all three subspecies of the bacterium (see details below). In each rat, one single screw was inserted into the right proximal tibia and peri-implant bone changes were monitored over time (post-operatively and at 3, 6, 9, 14, 20 and 28 days) with in vivo microCT. After 28 days, rats were euthanized and further evaluated through quantitative bacteriology. Three rats from the *S. epidermidis* group*,* and eight animals from the *C. acnes* cohort were processed for semiquantitative histopathological analysis.

### Bacteria and inoculum preparation

The *S. epidermidis* strain (Epi103.1) used is a clinical isolate available from the Culture Collection of Switzerland (CCOS1152). The *C. acnes* strains were also clinical isolates (Belfast, UK) and represented *C. acnes* subsp. *acnes* (type IA_1_ and IB), *C. acnes* subsp. *defendens* (type II) and *C. acnes* subsp. *elongatum* (type III).

The rats were inoculated via bacteria-contaminated screws prepared immediately prior to each surgery as previously described^9^. Bacterial stock cultures were stored at − 80 °C in 20% (v/v) glycerol. *S. epidermidis* was cultured on tryptic soy agar (TSA, Oxoid, Basel, Switzerland) or in tryptic soy broth (TSB, Oxoid) in ambient air at 37 °C. *C. acnes* were grown anaerobically on anaerobic blood agar (ABA, Oxoid) in a GasPak EZ System (BD Diagnostics, Allschwil, Switzerland). Broth culture of all *C. acnes* strains was performed in proteose peptone yeast (PPY) medium (Oxoid). Immediately before use, L-cysteine (0.75 mg/ml, Sigma Aldrich, Buchs, Switzerland) and sodium bicarbonate (1.5 mg/ml, Sigma Aldrich) were added to the PPY medium to maintain a reducing environment.

A *S. epidermidis* liquid culture in logarithmic phase was used (37 °C, 100 rpm), while for *C. acnes,* colonies grown for 7 days on ABA plates were used. For both species, bacterial suspensions were washed by centrifugation (2500 g for 10 min) and then resuspended in phosphate-buffered saline (PBS, Sigma Aldrich) or quarter strength Ringer's solution (BRAUN, Switzerland) with 0.75 mg/ml cysteine (QSRSc*, C. acnes)*. The threaded part of the screws were submerged for 20 min at room temperature, aerobically for *S. epidermidis,* or in an anaerobic box for *C. acnes*. *C. acnes*-colonized screws were transported to the operating room in an anaerobic box and all screws were implanted within a maximum of 30 min following preparation. Test screws were included within each experiment and quantitatively assessed for bacterial adhesion with the appropriate agar and incubation conditions.

### Animal welfare, observation and euthanasia

Skeletally mature, female, specific pathogen-free Wistar rats (24 weeks old), purchased from Charles River (Germany), were used in this study. Animal welfare was evaluated by the animal caregivers using a scoresheet developed in-house. The animals were scored twice per day until day 3, daily until day 7, then weekly until day 28. All rats were weighed post-operatively (day 0) and at day 3, 6, 9, 14, 20 and 28. The rats were euthanized via intracardiac injection of pentobarbital on day 28 under isoflurane anesthesia.

### Implant design and manufacturing

Custom-made (5 mm length, × 1.5 mm diameter) screws^11^ were machined from medical grade polyetheretherketone (PEEK) containing 20% (w/v) barium sulphate to impart radiodensity (RISystem AG, Davos, Switzerland). The screws were then cleaned by ultrasonication in a series of washes in isopropanol (Sigma-Aldrich), 70% ethanol (Sigma-Aldrich) and ultrapure MilliQ water (15 min each). All screws were autoclaved at 121 °C for 20 min before implantation.

#### Anesthesia and surgery

Anesthesia and surgery were performed as described previously^12^. In brief, after preoperative analgesia (buprenorphine 0.03 mg/kg, *s.c.* and carprofen 5 mg/kg, *s.c.*), the rat was anesthetized with isoflurane and the right tibia aseptically prepared. A 1 cm incision was made on the proximolateral aspect of the right tibia. A ø1.2 mm unicortical hole was drilled 2 mm distal to the growth plate, then tapped (2 mm outer ø/1.2 mm inner). After the sterile or colonized screw was inserted manually, the fascia and skin were closed in two layers using absorbable suture material (Monocryl and Vicryl rapid, Ethicon Inc., Cincinnati, USA; sizes 6–0 and 5–0, respectively). For postoperative analgesia, buprenorphine (0.05 mg/kg, *s.c.*) was administered every 12 h for 3 days and paracetamol (7 ml Dafalgan syrup/100 ml water; Bristol-Myers Squibb) was given via drinking water for a period of 7 days.

### Time-lapse in vivo MicroCT

MicroCT scans of the proximal tibia were acquired immediately postoperatively and at 3, 6, 9, 14, 20 and 28 days after surgery (VivaCT40, Scanco Medical AG, Bruettisellen, Switzerland). Anesthesia was induced and maintained with isoflurane during scanning. The tibia was pulled to full extension and fixed into the holder. The scanned region was 10 mm long, centered on the screw, with a ø25.6 mm field of view. The X-ray tube was operated at 70 kV voltage, 114 µA current with a 0.5 mm aluminum filter. 1000 projections were acquired over 180° rotation, with 220 ms integration time, resulting in a scan time of 21 min with a radiation dose of 200 mGy (previously shown to have no effect on bone turnover^13,14^). The total anesthesia duration was 25–30 min per scan. For each scan, 420 slices were reconstructed across an image matrix size of 1024 × 1024 voxels with a nominal voxel size of 25 µm.

### Image processing

**Figure 1:**
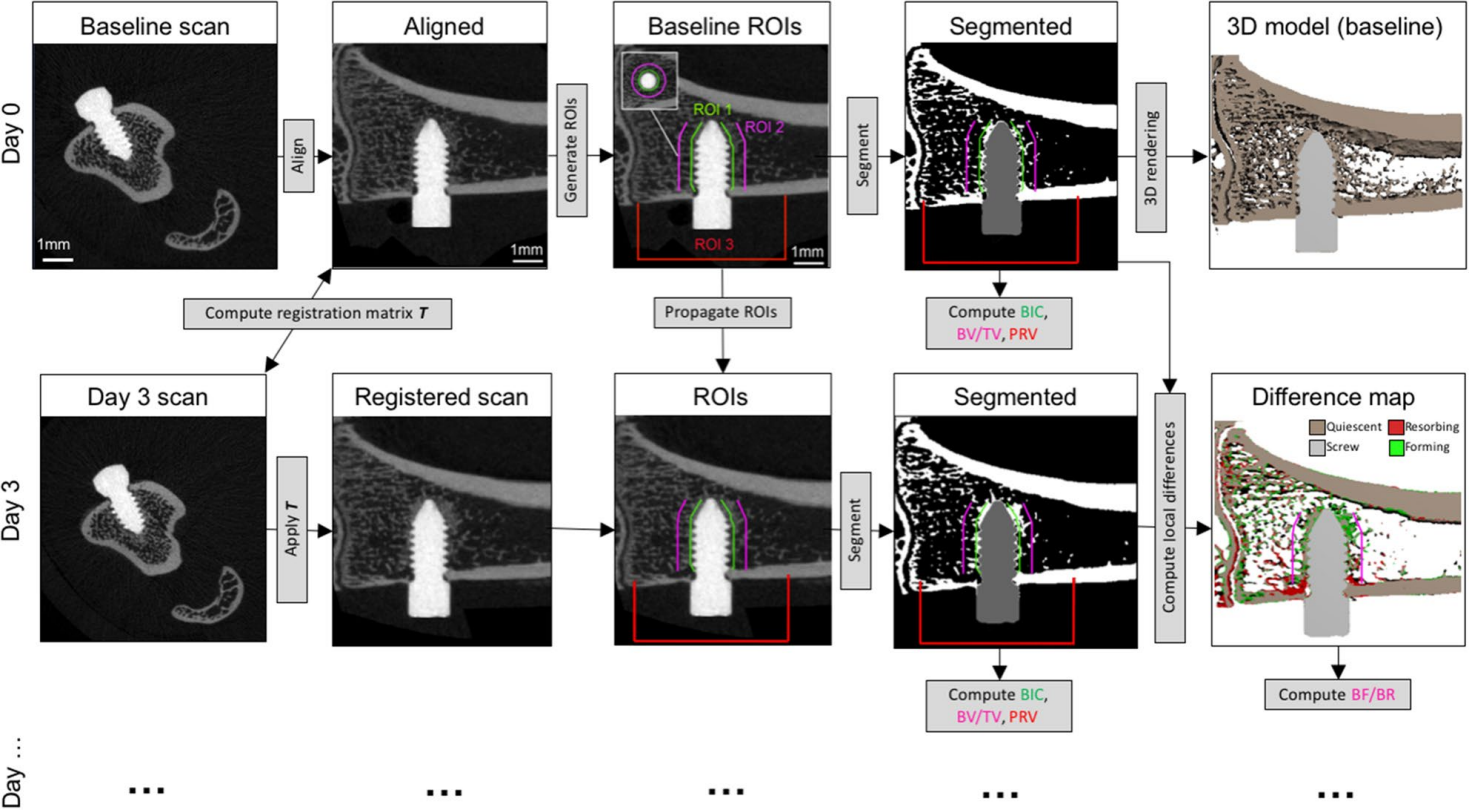
Schematic of the image processing workflow. The baseline scans were aligned to XY then subsequent scans are registered rigidly. The ROIs are defined in the aligned baseline scan. Two ROIs were automatically generated in the trabecular bone. ROI 1 at screw surface for bone-implant contact (BIC) and ROI 2 within peri-implant trabecular bone for peri-implant bone fraction (BV/TV), microstructure and changes (BF/BR). ROI 3 was automatically generated in the medial periosteal region of the tibia 2 mm proximal and distal of the screw head to evaluate the volume of periosteal reaction (PRV). The aligned scans were segmented using global thresholds for bone and PEEK. BIC, BV/TV, BMD and PRV were computed in the aligned images. Two consecutive segmented scans were overlapped to generate a difference map from which bone formation, resorption and quiescence are labelled and BF/BR quantified.

In order to standardize processing, postoperative scans served as baseline and were rotated so that the screw was aligned to the Y-axis and the tibia to the X-axis of the image using 3D rotation and resampled using linear interpolation (Fig. 1). The subsequent series of scans were aligned to baseline via rigid registration (with minimum 0.8 correlation coefficient) and resampled using linear interpolation. At this point all scans had been through rotation/interpolation filter and, therefore, Gaussian filters were not used before thresholding. The scans were segmented with a threshold of 580 mgHA/cm^3^ for bone and 1500 mgHA/cm^3^ for PEEK. The screw was dilated by one voxel after thresholding to prevent partial volume effect in the surrounding bone measurements. In the baseline scan, three regions of interest (ROI) were automatically generated: implant surface (ROI 1) defined as the volume within 75 µm from the threaded screw surface, was generated by a 3 voxel dilation of the screw minus the screw. Peri-implant volume (ROI 2), defined as the volume within 75-to-700 µm from the threaded screw surface, was generated by a 28 voxel dilation of the screw, minus the screw and ROI 1, and limited in Y to the screw thread position. Periosteal volume (ROI 3) was defined as the medial periosteal region 2 mm distal and proximal from the screw head. ROI 3 was generated by closing the segmented bone and screw, and inverting this object, to keep only the outside volume. This volume was intersected with a cube of 5 mm sides centered on the screw head, limiting de facto the ROI to the medial side where the screw head protrudes from bone (Fig. 1).

Bone-implant contact (BIC) was computed within ROI 1 by automated voxel-counting of the segmented bone; bone fraction (BV/TV) was computed within ROI 2 using standard methods for characterization of bone microstructure^7^. Bone formation (BF) and bone resorption (BR) were computed by mapping the segmentation differences between two subsequent registered scans^8,15,16^ within ROI 2 (Fig. 1). This method is sometimes referred to as “dynamic histomorphometry”^17^. For periosteal reaction, tissues were segmented within ROI 3 using a 375 mgHA/ccm threshold (periosteal reaction being less calcified than bone). This threshold value was defined empirically from the histograms of clearly identifiable periosteal reactions. Periosteal reaction volume (PRV) was then simply computed by voxel counting. Image processing algorithms were developed with EasyIPL v1.0.2 (available at easyipl.com), a high-level library of macros using the scanner software (Image Processing Language, IPL V5.42, SCANCO Medical) and OpenVMS DIGITAL Command Language, DCL V8.4-1H1, Hewlett Packard).

To determine the sensitivity of dynamic histomorphometry in this model, four rats were scanned seven times each after euthanasia (with complete retrieval and replacement into the holder between each scan). In theory, the series of identical images should lead to identification of quiescent bone but, in practice, image noise or stack misalignment can generate changes between time points. The dynamic histomorphometry procedure was applied to the cadaveric image series. Variations of BIC and BV/TV values over time were below 4%. BF and BR measured from registered repeated scans were 0.33 ± 0.17 mm^3^/day and 0.3 ± 0.15 mm^3^/day, respectively. The remodeling ratio BF/BR was 1.1 ± 0.15. Thus, for the reported evaluations described here, BF/BR values within this range are considered quiescent and BF/BR differences < 0.15 were deemed not significant.

#### Histological processing and analysis

For comparison with a microCT image, the specimens were fixed and embedded in methyl methacrylate, as previously described^11^. One section from each specimen was surface stained with Giemsa-Eosin for tissue morphology. Another section was stained for Brown-Brenn, a special staining for gram-positive bacteria. We manually identified the gross position and orientation of the histological section in the corresponding microCT image based on the bone and screw shapes. Anatomical landmarks in the histological slice were then located visually in the corresponding microCT scans. The spatial coordinates of these features were used to rotate the scan spatially and resample it in the plane of the histology slide.

### Bacteriology

After euthanasia, the limbs were dissected and the screws and bones were collected in separate, sterile containers containing 5 mL PBS or QSRSc, as previously described^11,18^. Any soft fibrous tissue overlying the protruding head of the screw was also collected in separate sterile containers. The number of bacteria adhering to the *S. epidermidis-*inoculated screws was determined following sonication for 3 min and vortex mixing for 10 s, before performing serial dilutions and viable bacteria counts on 5% (v/v) horse blood agar (Oxoid)^9,10^. The entire tibia from each animal was then mechanically homogenized in PBS (Omni Tissue Homogenizer and Hard Tissue Homogenizing tips, Omni International, Georgia, USA) and the quantity of bacteria associated with bone similarly determined by colony counting^11,18^. Soft tissue samples were also processed in the same manner. All agar plates were incubated for 24 h at 37 °C and all growth checked for contamination or signs of coinfection^11,18^. Samples from *C. acnes* inoculated rats were processed in the same manner, except that samples were processed in QSRSc and incubated anaerobically for 7 days. Quantitative CFU data is presented as a sum of all three samples (soft tissue, bone, and implant).

To confirm that the bacteria growing in culture-positive samples from *S. epidermidis-*inoculated rats were actually infected with the iatrogenically inoculated strain, a random amplified polymorphic DNA (RAPD) PCR assay was performed, with positive (inoculated strain) and negative (water only) controls included^19^.

### Statistical analysis

Data is expressed as mean ± SD, except for histopathological results, which is presented as median values. Historical data for twelve *S. aureus* inoculated animals were used for comparison^11^. The *S. aureus* data were re-analyzed with the latest algorithms in the newly defined ROIs to have comparable outcomes between groups, as confirmed when comparing new and historical sterile animals (data not shown). The time series curves are shown as mean ± SEM for the measured data points. They were modelled using a general additive mixed model (GAMM) approach with a smoothing on time; a post-hoc test was run to determine statistical significance between groups using the *gam_wald* function of the *itsadug* R package^20^. The 95% confidence intervals (CI) of the fitted models were overlaid with transparent colors. Time periods where CIs do not overlap show when the series were statistically different from each other*.* For single time point parameters (bacteriology, histology), ANOVA was used to determine significance and Tukey post-hoc to test differences between groups. Significance levels of p < 0.05 were considered statistically significant (*). All analyses were performed using the R software program (version 3.3.3)^21^.

## Results

### Animal welfare

Of the 38 rats which underwent surgery, 34 are included in the final analysis. The four exclusions included three rats that had surgery in a pilot study to determine the optimal inoculation dose for *C. acnes* (data not shown) and one was euthanized on day 6 due to a breathing problem unrelated to the surgery or infection. One rat was found dead in its cage on day 20 without any indications of sepsis or severe local infection, but the data acquired to that point were included in microCT evaluations. No rat was euthanised due to reaching predefined thresholds in animal welfare score. The total numbers per group at study completion were: sterile n = 6; *S. epidermidis* n = 6; *C. acnes* IA_1_ n = 5; IB n = 6; II n = 6; III n = 5. Otherwise, all rats tolerated the surgery and CT scanning regime, and did not approach our predefined exclusion criteria. Overall, rats from all groups lost weight in the first few days after surgery. However, by completion of the observation period, 5/6 rats in the sterile group, as well as 2/6 *S. epidermidis* and 13/22 *C. acnes-*inoculated rats had either recovered or exceeded their initial weight (data not shown). No rat lost more than of 5% of initial body weight at euthanasia.

### Quantitative bacteriology

Quantitative bacteriological evaluation of the number of adherent bacteria on the screws at implantation revealed that the *S. epidermidis-*inoculated rats received an approximate dose of 1.3 × 10^6^ CFU per screw (range 9.8 × 10^5^–1.6 × 10^6^), whilst the *C. acnes-*inoculated rats received approximately 1.0 × 10^6^ CFU (range 3.1 × 10^5^–3.8 × 10^6^).

**Figure 2:**
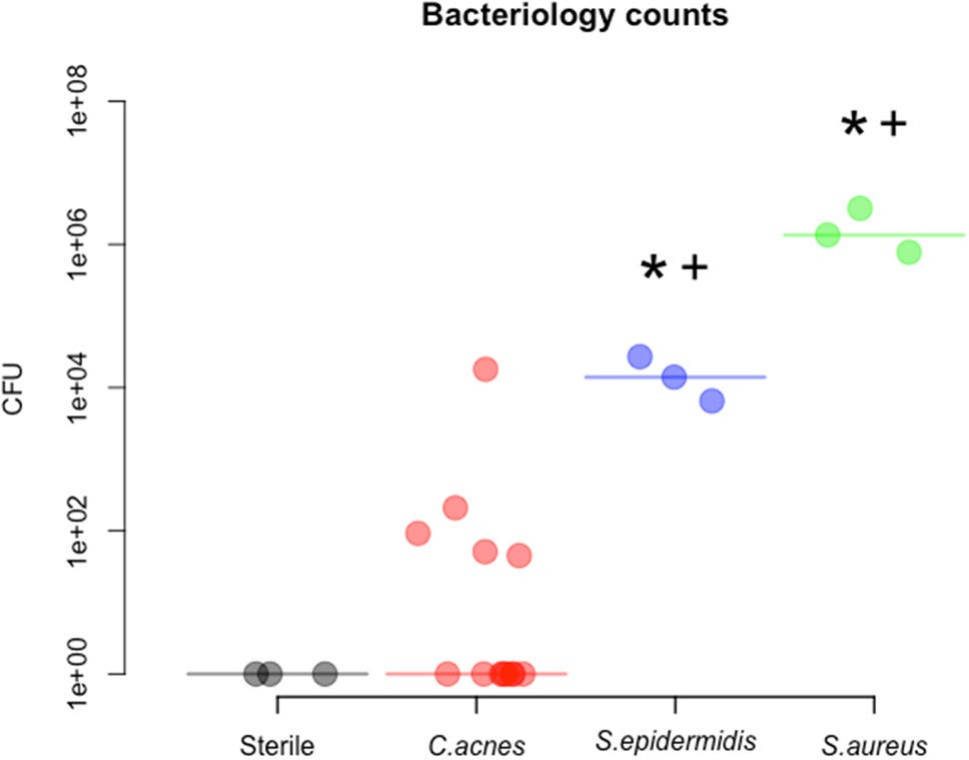
Bacteriological outcome of infection. After euthanasia at day 28 post-implantation, rats were dissected and separate CFU counts were performed on the implant, overlying soft tissue and the bone (sum of all locations presented here). Data shown is from 3/3 animals per group, with the exception of *C. acnes* (pooled) with 14 animals in total. Historical *S. aureus* results are also shown for comparison. Horizontal lines indicate median values per group. ANOVA analysis: effect of bacteria strain p < 0.0001; post-hoc: * significantly different from the sterile group; ^+^ significantly different from *C. acnes* group. Due to the logarithmic nature of the y axis, culture-negative samples were assigned an arbitrary value of 1.

Data for animals submitted for quantitative bacteriology at euthanasia are shown in Fig. 2. All sterile control animals (3/3) were found to be culture-negative, whilst 3/3 *S. epidermidis*-inoculated animals were culture-positive and 5/14 *C. acnes-*inoculated rats were culture-positive. Within the *C. acnes* subgroups*,* 2/3 type IA_1_, 0/3 type IB, 0/3 type II and 3/5 type III inoculated rats were culture positive. A more detailed presentation of bacteriological outcome, including individual results in soft tissue, in bone and on the implant is shown in supplementary Fig. 1.

### Infection development assessed by microCT

**Figure 3:**
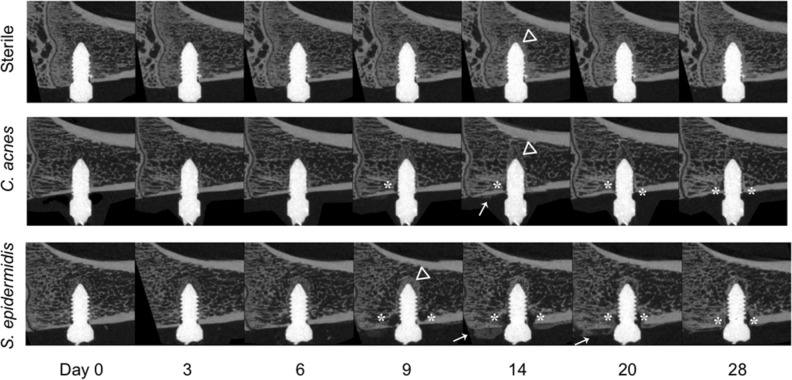
Longitudinal in vivo microCT series. Animals in the sterile group showed a steady bony integration of the screw until day 14 (triangles), followed by remodeling. Animals in the *C. acnes* group showed reduced bone growth and some contact loss (asterisks), which appeared near the cortex. Animals in the *S. epidermidis* group showed both bony integration near the screw tip, and bone resorption near the screw neck. Periosteal reaction (callus formation; arrow) was sometimes visible in the *C. acnes* group, and a marked periosteal reaction was visible in the *S. epidermidis* group, at day 14 and 20. Selected images were chosen on the basis of having BV/TV area under the curve closest to median for their respective group.

The sterile, *C. acnes* and *S. epidermidis* groups displayed different patterns of bone changes around the implant, as shown in still images in Fig. 3. In all animals of the sterile group, new bone progressively formed until the screw was completely integrated at day 14, after which time the bony tissue surrounding the screw was progressively remodeled. In the *C. acnes* group, some osteolysis of cortical bone and contact loss at the screw was evident, although no extensive osteolysis was observed. In the *S. epidermidis* group, bony integration did not occur in the majority of animals, with cortical and trabecular osteolysis evident from day 6–9 onwards.

**Figure 4:**
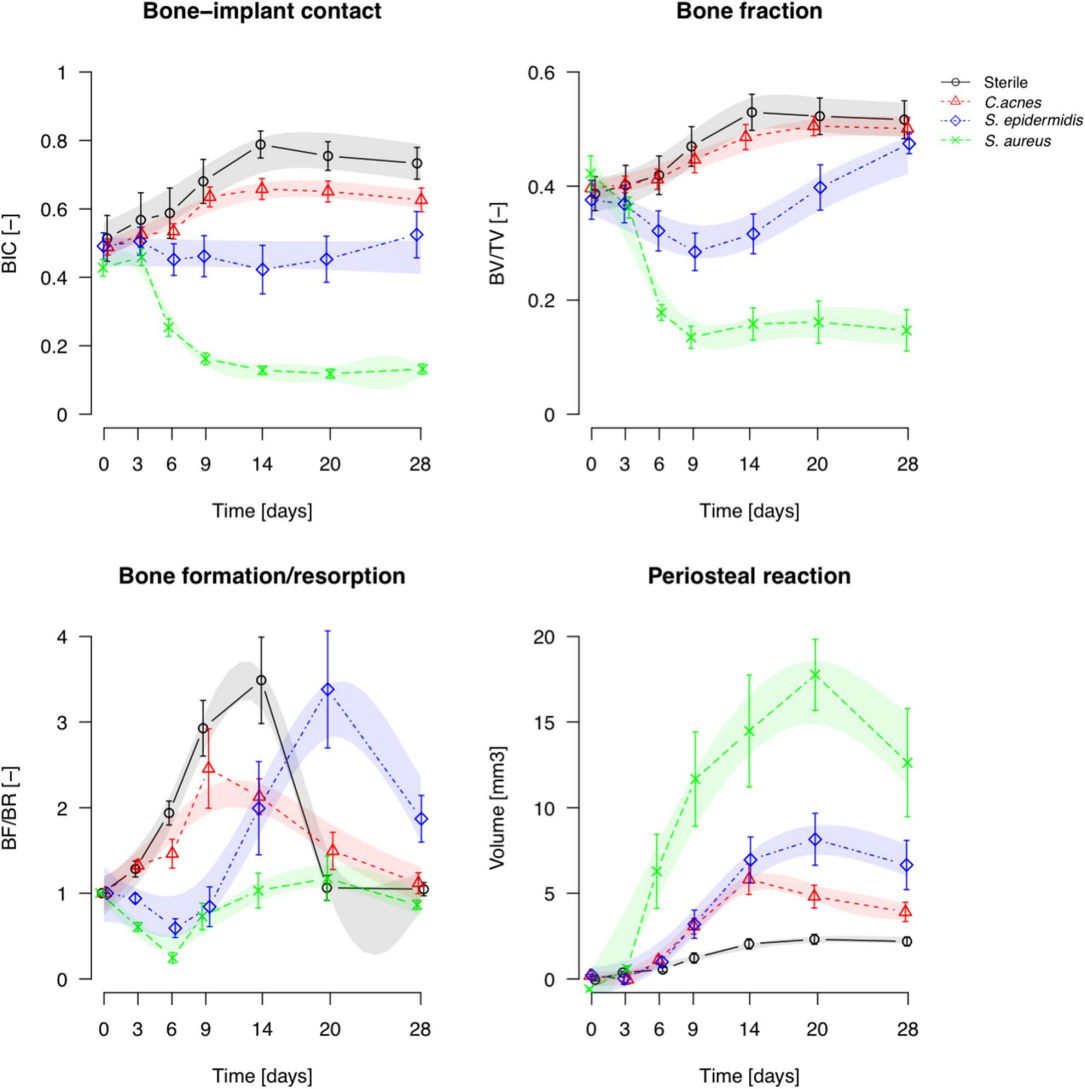
Quantitative analysis of longitudinal microCT assessment of bone parameters. Data shown are mean ± SEM, transparent color shades represent the Bayesian GAMM model 95% confidence intervals (CI). Statistical significance is extrapolated from the confidence intervals: when the CIs of two groups do not overlap, they are considered significantly different during that time period. (*S. aureus* is a re-analysis of historical data from^11^).

Quantitative analysis of the longitudinal microCT data is shown in Fig. 4. Historical data for *S. aureus*^11^ (reanalyzed with the updated protocols) are included for comparison. Animals receiving sterile screws displayed a steady increase in BIC and BV/TV (bone fraction) from day 0 until 14 days post-op, followed by a very slight decline. Animals in the *C. acnes* group showed similar patterns, but with significantly lower BIC on day 14, and a significant delay in reaching peak BV/TV (from day 14 in the sterile group to day 20 in the *C. acnes* group). *S. epidermidis*-inoculated animals had no increase in BIC, which was significantly different from those receiving a sterile screw from day 6 onwards. BV/TV was temporarily reduced in the *S. epidermidis*-inoculated animals but recovered to similar values as the sterile and C*. acnes*-inoculated animals on day 28. The *S. aureus* group displayed a marked reduction in both BIC and BV/TV within 9 days, and minimal (if any) recovery at later stages.

Bone remodelling ratio (BF/BR) also showed differences between the groups, as shown in Fig. 4. Bone formation and bone resorption are presented as separate measures in Supplementary Fig. 2. *C. acnes* displayed a lower remodelling ratio compared to sterile groups, whilst for the *S. epidermidis* group this was delayed relative to the sterile group. In contrast*,* the *S. aureus* group displayed minimal remodelling changes over time. Periosteal reaction tended to increase over time in all groups until days 14 to 20, with a decline thereafter. Peak periosteal reaction was highest for *S. aureus*, with subsequent further reductions for *S. epidermidis*, then *C. acnes* and, finally, the sterile group.

### Semi-quantitative histopathology and visual validation of microCT

**Figure 5:**
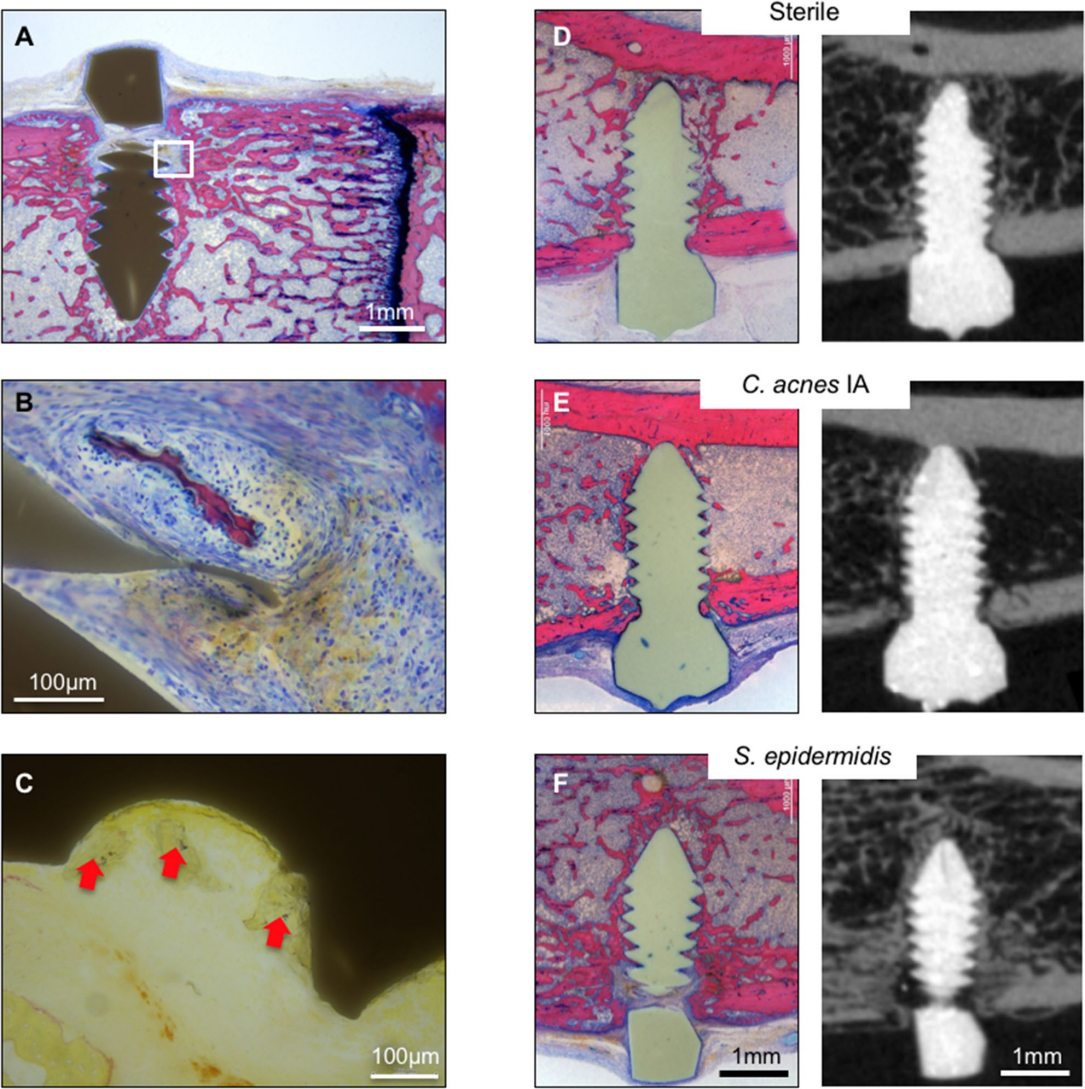
Histopathological observations of infection and comparative microCT-and histology sections. Left-hand side: microphotographs of the right proximal tibia with an *S. epidermidis* inoculated screw. (**A**) Overview (scale bar: 1 mm). Note the microabscesses around tiny bone sequesters (white square). (**B**) Higher magnification of white square in A; (scale bar: 100 µm). Note the presence of small microabscesses, characterized by a thin layer of fibroblasts surrounding the granulocytes present around centrally located necrotic bone sequesters (as determined by the presence of empty osteocytic lacunae), and the irregular, sculptured surface, indicating osteoclastic bone degradation. Neither of these features would be visible on microCT (MMA-embedded, Giemsa Eosin-stained thick-section). (**C**) Intermediate magnification of tissue near the screw thread (scale bar: 100 µm). Note the bluish-stained BB-positive bacteria (red arrows). Right-hand side: side-by-side comparison of matched histological (Giemsa Eosin stained; scale bar: 1 mm) and microCT sections. Note bony integration of the screw with surrounding bone in the sterile group. The C. acnes group displays similar integration in the medullary area, although there is evidence of cortical osteolysis indicative of ongoing infection. The S. epidermidis group displayed reduced integration compared to the sterile and C. acnes groups, with some apparent cortical osteolysis in the vicinity of the screw. MicroCT images shown were selected to match the corresponding histological section. Since histology provides only a single section, appearances may not reflect the quantitative observations provided by MicroCT, highlighting the value and importance of performing 3-dimensional imaging.

Representative histological slides for each group are shown in Fig. 5. The *S. epidermidis-*inoculated groups that were confirmed culture-positive in all cases displayed minor signs of infection in the histological sections, such as reduced direct osseous integration and formation of fibrous tissue especially at the cortical area (Fig. 5A). Microscopically, reduced osseointegration was accompanied by the presence of low amounts of Giemsa-positive, coccoid microorganisms (Fig. 5C). In general, the three *C. acnes* groups displayed a similar appearance, with unaffected osseointegration and no signs of infection. Seven histology sections were successfully identified in the corresponding microCT scans using anatomical landmarks (Fig. 5D–F).

Right-hand side: side-by-side comparison of matched histological (Giemsa Eosin stained; scale bar: 1 mm) and microCT sections. Note bony integration of the screw with surrounding bone in the sterile group. The *C. acnes* group displays similar integration in the medullary area, although there is evidence of cortical osteolysis indicative of ongoing infection. The *S. epidermidis* group displayed reduced integration compared to the sterile and *C. acnes* groups, with some apparent cortical osteolysis in the vicinity of the screw. MicroCT images shown were selected to match the corresponding histological section. Since histology provides only a single section, appearances may not reflect the quantitative observations provided by MicroCT, highlighting the value and importance of performing 3-dimensional imaging.

### Analysis of C. acnes subspecies

**Figure 6:**
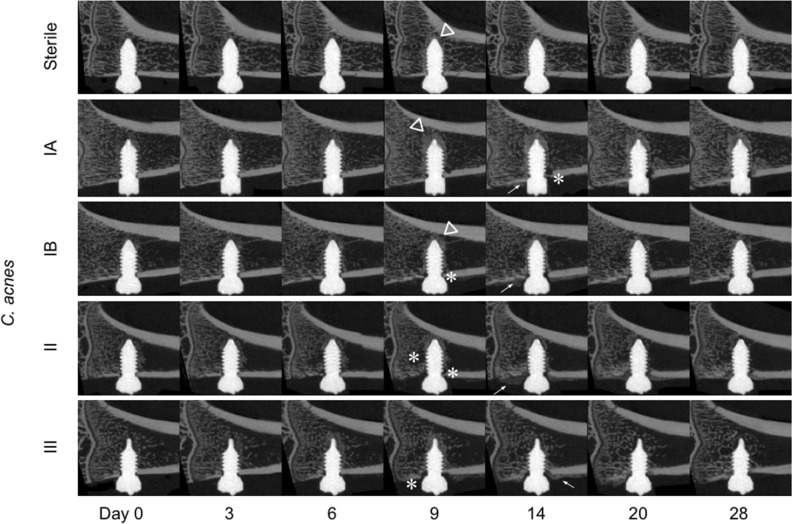
Longitudinal microCT series of *C. acnes* subspecies. The infected series have more visible changes in peri-implant bone than the sterile series, with increased bony integration (triangles), trabecular and cortical osteolysis (asterisks) and periosteal reaction (arrows). Selected images were chosen based on having BV/TV area under the curve closest to the median for their respective group.

**Figure 7:**
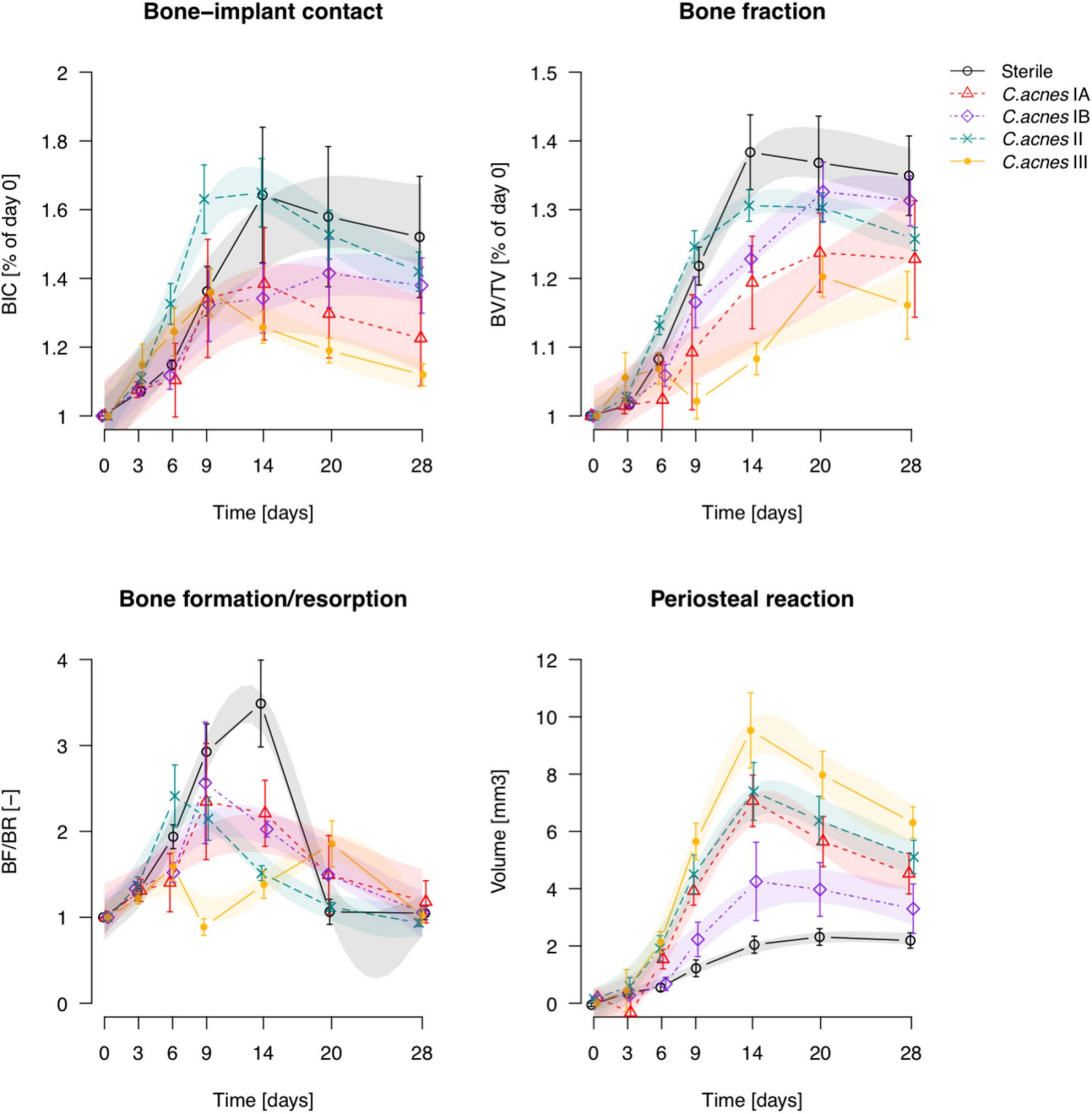
Quantitative analysis of longitudinal microCT assessment of bone parameters for *C. acnes* subspecies. Infected animals display significantly lower BIC compared to the sterile animals from day 14 onwards in all strains, with the exception of type II.

An analysis of *C. acnes* subsp. *acnes* (types IA_1_, IB), *C. acnes* subsp. *defendens* (type II) and *C. acnes* subsp. *elongatum* (type III) was performed in order to test whether the model was sufficiently sensitive to differentiate between bacterial subspecies and strain types. Upon visual inspection, the microCT series of each *C. acnes* group appear different from the sterile group animals. Osteolysis of peri-implant cortical bone was visible in all the type II and III inoculated rats from day 6–9. Otherwise, no visually obvious distinctions could be made (Fig. 6). In quantitative terms (Fig. 7), animals infected with all strains, with the exception of type II, showed lower BIC and BV/TV values compared to the sterile group from day 14 onward. All inoculated animals showed lower BF/BR compared to the sterile group. The clearest distinction between strains was measurable in terms of periosteal reaction, with each showing a distinct periosteal volume curve (Fig. 7).

### Distinguishing between active and resolved C. acnes infection

Of the 14 *C. acnes*-inoculated rats submitted for bacteriological assessment, only five were determined as culture-positive at euthanasia (two with type IA_1_, and three with type III). To determine whether there was an observable difference between culture-negative (i.e. those that were inoculated but resolved the infection over the course of the study) versus culture-positive rats (inoculated and had an active infection at euthanasia), we performed an additional analysis of the *C. acnes* phylogroups at day 28 between culture-positive (n = 5) and culture-negative (n = 9) animals.

**Figure 8:**
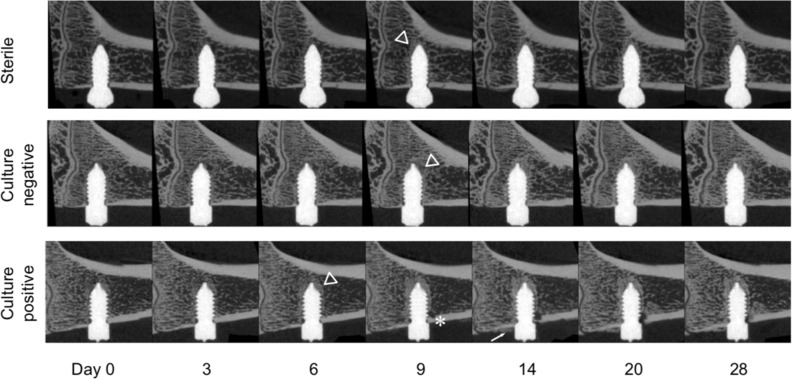
Longitudinal in vivo microCT series of active and resolved *C. acnes* infections. Sub-analysis of active (culture-positive) and resolved (culture-negative) *C. acnes* infections over the 28-day experimental duration. Culture-negative series shows bone encapsulation (triangles) of the screw similar or superior to that of the sterile group. Peri-implant bone resorption (asterisks) and periosteal reaction (arrows) are observed in the animal with active infection. Selected images were chosen based on having a BV/TV area under the curve closest to the median for their respective group.

**Figure 9:**
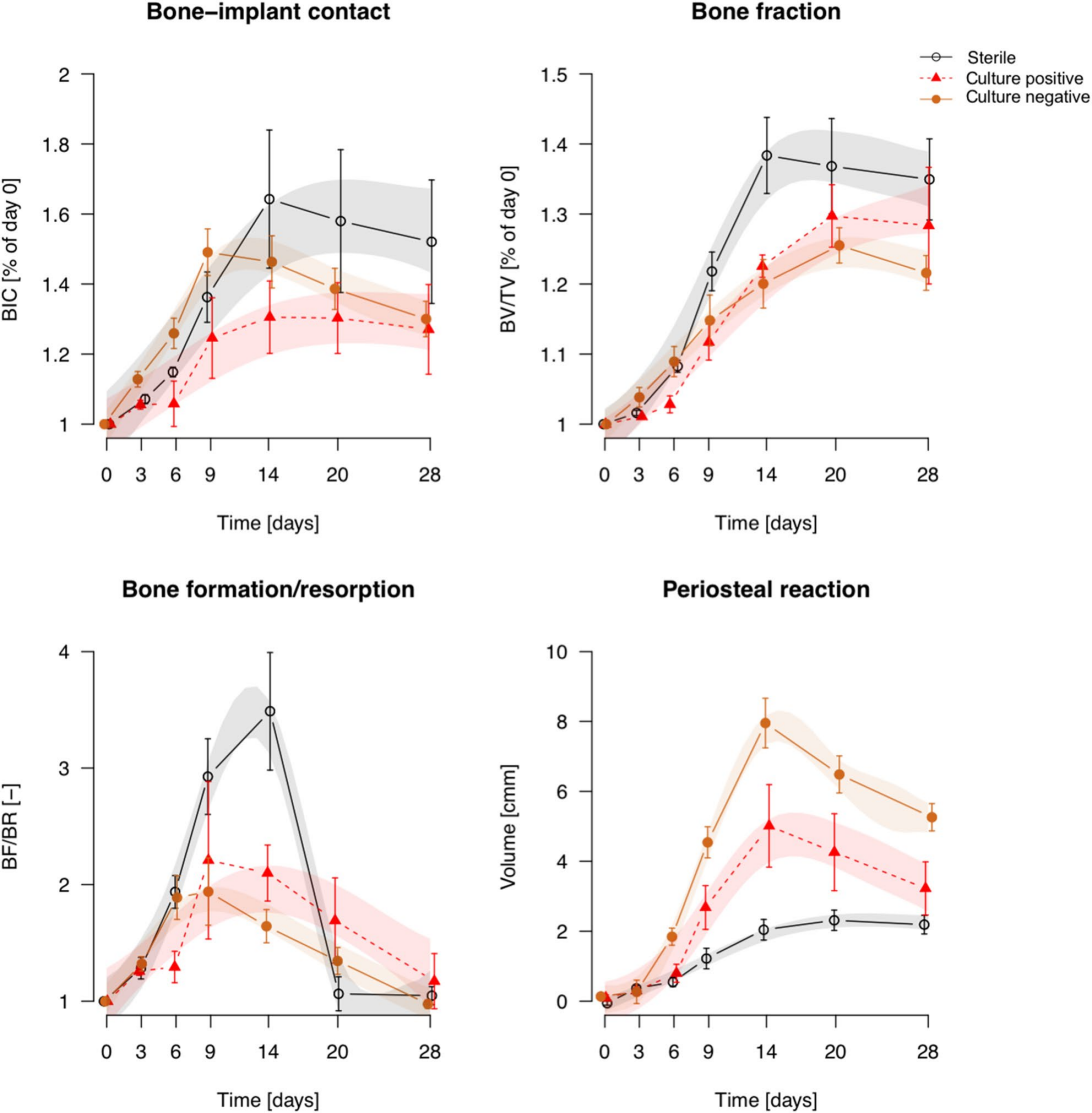
Quantitative analysis of longitudinal microCT assessment of bone parameters for active and resolved *C. acnes* infections. Analysis of active (culture-positive) and resolved (culture-negative) *C. acnes* infections over time. Both culture-positive and culture-negative animals displayed similar responses in bone parameters over time compared to sterile animals, with the exception of periosteal reaction. Periosteal reaction was markedly increased in culture-negative animals compared to culture-positive animals.

Visually, osteolysis was detectable in the microCT series for only some culture-positive animals, but not in the culture-negative animals or sterile animals (Fig. 8). Quantitatively, the difference between culture-positive and culture-negative animals was only detectable at the screw surface and in the periosteal space (Fig. 9). Culture-positive animals had reduced BIC values between day 9 and 20 compared to culture-negative, but no difference was detected between groups at day 28. No clear distinction between culture-positive and culture-negative animals was obvious for BV/TV or BF/BR rates but, interestingly, the periosteal reaction in culture-negative animals was significantly increased over culture-positive animals from day 9 to 28 (Fig. 9). At most time points, both culture-positive and culture-negative animals displayed different patterns compared to the sterile animals.

## Discussion

The clinical diagnosis of acute ODRI is often simple, with obvious clinical symptoms of inflammation such as swelling, redness, pain, and clear radiographic changes such as osteolysis or periosteal reaction. In contrast, the clinical signs of subclinical infection may be subtle, and radiographic changes may only appear after several months, if at all, making diagnosis a challenge and delaying onset of treatment. The goal of this study was to evaluate dynamic, high resolution, quantitative CT imaging in a rat model of subclinical ODRI caused by *S. epidermidis* and *C. acnes* and compare outcomes with sterile and historical *S. aureus*-inoculated controls.

The animal model together with the analytical protocol developed in this study has proven to be suitable for evaluating peri-implant bone changes in great detail without animal welfare concerns. The infection-related changes are localized to the surgical site and appear well tolerated by the rats; the repeated CT scan protocol is simple and standardized, and the model runs over 28 days, thereby encompassing time points for early detection, but also later time points where the sequelae of an infection may be observed. The model is an evolution of our past work using titanium^22^ or titanium coated peek screws^11^, both of which resulted in metal artefacts and/or non-homogeneous coating. In the present iteration of the model, barium sulfate-enhanced PEEK gives good image quality and image processing is easily automated, making the model suitable for longitudinal studies. The use of PEEK also has clinical relevance, since it is increasingly used in orthopedic medicine and we have shown that the progression of infection around PEEK devices is essentially identical to titanium in a mouse model^23^. Using this model, non-infected rats display an uninterrupted progression of implant integration and bone remodeling, peak bone formation at day 14, and minimal periosteal reaction. The infection-challenged rats displayed different degrees of bone changes that varied over time, and between species and ongoing versus resolved infection. Differences between species were detected that were far from obvious from visual observation of still microCT images, illustrating the added value of merging 3D data from subsequent timepoints, producing inherently richer 4D data.

For *S. aureus*, the predominant agent of acute ODRI, marked bone changes were seen in rats across each observed parameter when historical data^11^ were reanalyzed with the latest algorithm. All *S. aureus*-inoculated animals were culture-positive with a higher CFU count than the other species tested; virtually no peri-implant bone was present after 6–9 days and periosteal reaction was greatly increased compared with all other species. Our understanding of bone-pathogen interactions and infection-induced bone changes come largely from studies on *S. aureus,* which can change bone remodeling^24^, trigger bone destruction through osteoclasts^25^, release osteolytic proteins^26^ and even trigger immune responses that activate pro-osteogenic pathways as well as bone destruction^27,28^. The dynamic histomorphometry data presented here confirms that the bone loss in an *S. aureus* infection progresses significantly beyond the implant surface and results in pronounced osteolysis caused by inflammatory processes around the implant, with formation of (micro)abscesses and fibrous tissue.

The focus of the present work was, however, to determine the comparative effects of subclinical infection. Firstly, the *S. epidermidis*-inoculated rats were all culture-positive at euthanasia and showed moderate bone changes, such as peri-implant osteolysis and periosteal reaction, and not the acute and rapid bone loss associated with infection caused by more virulent bacteria such as *S. aureus*^11^. Peak bone formation was delayed to day 20 compared with day 14 in non-infected rats, and significantly more periosteal reaction was measured than in non-infected control animals. Studies into the mechanisms of *S. epidermidis*-induced bone loss are comparatively rare, though it is known to retain fewer virulence factors and is considered less pathogenic and thus less likely to induce significant bone loss^29^. The data presented here confirms that there is not only an absence of bone loss over time compared to *S. aureus*, but also less marked bone changes around the implant, which were clearly detectable using this approach (e.g. positive BV/TV and BF/BR ratios, lower periosteal reaction).

The second opportunistic pathogen in focus was *C. acnes.* In this group, the rats did not show persistent infection, in marked contrast to both *S. epidermidis* and *S. aureus-*inoculated rats. Overall, the bone formation ratio evolution in the *C. acnes* group was comparable to sterile rats, and periosteal reaction was milder compared to *S. epidermidis*. Interestingly BF/BR curves were similar between culture-positive and culture-negative *C. acnes* inoculated rats, suggesting that perhaps the immune response to the bacteria (dead or alive) may be the dominant factor, rather than bacterial virulence factors which would likely require viable bacteria for a persistent effect. Consistent with this, heat-killed *C. acnes*, as well as soluble polysaccharides extracted from the cell wall of *C. acnes*, have been shown to induce dendritic cell maturation to promote type 1 responses and pro-inflammatory cytokine production both in vitro and in vivo^30–32^. A past immune reaction can also influence bone homeostasis^33^. Culture results at each time point would shed further light on whether this effect was perhaps due to the inability of *C. acnes* to reliably induce a persistent infection, and thus we are merely observing the effect of host clearance of the infection at different time points. Importantly, the only bone-related difference seen amongst *C. acnes* were observed in BIC, which is measured at the bone-screw interface (rather than bone fraction (BV/TV) or bone remodeling (BF/BR), which are evaluated deeper into the peri-implant bone). This suggests that *C. acnes*-induced bone responses may be linked with persistence on the surface of the device rather than invasive infection of the bone. Clinical studies into *C. acnes* have also shown that the bacteria tend to be found preferentially attached to the implanted device rather than in tissue samples^34^, thereby supporting the clinical relevance of this model and our findings.

A striking feature of the *C. acnes* group was the inability to consistently cause an infection. Culture conditions for these facultative anaerobes are more challenging than for the staphylococci also used in the study, and this is true for both inoculum preparation and post-mortem culture. All preparation and transportation techniques were performed, where possible, within an anaerobic environment. Our culture-positive results for both screws and post-mortem tissues indicated that the protocols were sufficient to detect a potential presence of bacteria. Other studies have shown a successfully established infection by this organism, both in our laboratory in rabbits, and in others using rats^35–37^. In any case, our data reveals that, despite a lack of persistent infection, some bony changes do occur and these patterns vary over time and across strains. This indicates that these bacteria can induce bone changes regardless of their ability to cause a persistent infection.

Looking across all inoculated species, periosteal reaction seemed to vary the most between groups. Periosteal reaction is typically present in more than 50% of the cases of acute, sub-acute or chronic osteomyelitis in humans^38^, but it is not specific to infection; it can also be caused by osteosarcoma, arthritis, local inflammation or mechanical stress^39,40^. In our study, periosteal reactions were greatest for *S. aureus* and reduced for *S. epidermidis* and *C. acnes,* with limited responses observed in the sterile group, thereby aligning with our pre-existing concepts of bacterial virulence and our bacteriological culture results in this model. Periosteal reaction was also present in culture-negative *C. acnes* rats, further suggesting that the link between periosteal reaction and infection is the immune reaction to bacterial presence, and not necessarily an active infection.

As an innovative imaging technique, the time lapse approach to monitor bone changes is an advance over past work in the field. MicroCT is frequently used as a stand-alone endpoint measure for preclinical studies of bone biology and bone pathology^41^ including bone infections^25,28,42^. Tracking changes in bone from a series of scans was first introduced in 2004 by Waarsing et al*.*^8^, but the idea of comparing radiographic longitudinal data to measure rates of bone changes was proposed as early as 1980^43^. The technique, sometimes named "dynamic histomorphometry"^17^, has predominantly been applied to study the bone changes following estrogen deprivation, loading and unloading^15,44,45^ and also implant integration^46–48^, but has not, to the best of our knowledge, been applied to the study of ODRI. Longitudinal microCT assessment of osteomyelitis is rare. Carlsson et al*.*^49^ studied the progression of *Mycobacterium marinum* infection in the mouse tail using, amongst others, microCT, but only reported bone volume and 3D images without image registrations. Li et al*.* studied the development of ODRI in a mouse model using post-mortem microCT at various time points, but did not assess quantitative changes over time^50^. Others have used a combination of optical imaging and microCT to monitor infection^51^, which overlays in a multimodal approach, bacterial presence (*S. aureus)*, immune responses and bone changes. It would be interesting to combine dynamic histomorphometry with an optical imaging method such as bioluminescence^51,52^ with the objective to compare the timings of both observations and also spatially correlate the resulting observations.

Of course, longitudinal time-lapsed microCT imaging does not come without limitations. Repeated anesthesia and exposure to ionizing radiation can have detrimental effects on the animal’s health, therefore the number of scans and the dose was minimized. However, it was shown previously that in rats the proximal tibia microstructure is unaffected after 5–8 weekly scans with doses up to 939 mGy^53^, and we showed previously that the osseointegration is also unaffected using the settings used here^22^. Sensitivity testing in repeated cadaver scans was limited to ± 0.15 BF/BR, and sensitivity is probably lower (i.e. larger minimal detectable change) in live animals, the scanning of which is more prone to noise and artifacts. The limited sensitivity can also be due to the relatively large voxel size (25 µm), the segmentation inaccuracies resulting from image noise, to partial volume effect or to registration inaccuracies, which can happen with multi-stack in vivo scans that are exposed to misalignments between stacks. The use of stack alignment methods could improve the quality of registration considerably in the future^54^, but at the cost of an increased radiation dose to the study animals. However, multi-stack misalignment is probably not a direct source of errors in our microstructural outcomes, since ROI 1 and ROI 2 are completely within the first stack. Concerning BIC, the thinness of ROI1 could make the measurement prone to errors, potentially metal artifacts and partial volume effects. We reduced drastically the metal artifacts problem by using contrast enhanced PEEK; its density is low enough that we did not observe any streaks, typical of metal artifacts. We used relatively large voxel size to improve the signal-to-noise ratio while reducing radiation dose, to the risk of missing very small bone changes. We also prevented partial volume effects with dilating the screw by one voxel after thresholding it and defining ROI1 after that voxel. In terms of validation of BIC it has been demonstrated that correlations of osseointegration between 3D microCT data and 2D histology data is limited because of incompatible dimensionalities^55^. Instead, here the validation was done in a slice-matched approach, where the microCT data is resampled exactly in the orientation of the histology slide and only one 2D slide is used. Nevertheless, 3D/2D registration remains technically challenging^56^. In our sample, we could only find a limited number of matching slices in which we could see similarities, supporting the concept that microCT provides enough data at earlier time points, thereby rendering animal sacrifice at intermediate time points unnecessary and difficult to justify.

Translation of rodent studies to humans is always a challenge, although it has been shown that osteomyelitis‐related bone changes are consistent among animal models^57,58^, and are similar to those seen in human patients^59^. Furthermore, concerning translation of the technology described here to human diagnostic imaging, the resolution of clinical CT is lower than microCT but is improving constantly, so one can expect that clinical dynamic histomorphometry may become a reality in the near future. The use of different implant materials, generally causing more artifacts at the bone-implant interface, is another limitation. It can also be expected here that advances in technology and image processing may enable similar evaluation protocols in human diagnostic imaging. To further add value to the approach, the method could be tested with high-resolution peripheral quantitative CT (HRpQCT) scans for peripheral infections, since the resolution of HRpQCT (60 µm) is sufficient to monitor bone changes in fracture repair or in joint degeneration^60,61^.

With the newly described model and analytical approach, we see numerous opportunities for further advancement of the model. Since the model is sufficiently sensitive to differentiate between infecting pathogens, it could be used in future to test/optimize infection treatments such as the testing of dual functional coatings (antimicrobial and osteoinductive), to promote bone regeneration as well as treating an infection. It could also be a useful measure of the extent of infected tissue to help identify regions for debridement. In theory, a library of patterns could be built, including other less frequent bacteria that can also affect bone^52,53^ and other bone diseases such as osteosarcoma, in order to have an effective diagnostic tool.

## Conclusion

Early detection of osteomyelitis is essential if appropriate therapy is to be started before bone necrosis. Time-lapsed microCT, the combination of spatial and temporal CT data, is a potent imaging method for this as it increases the sensitivity for small bone changes and, therefore, may enable the detection of signs related to potential infections earlier than “static” methods. In this rat model, dynamic histomorphometry could detect differences between bacterial species, and between culture-positive and culture-negative specimens. For research applications, monitoring of a single animal over time helps to reduce animal numbers, and could be used in combination with bioluminescence techniques to have a global overview of the development of ODRI.

## Supplementary information

Supplementary information.

Supplementary information.

Supplementary information.
