# Supplementary material for: Longitudinal time-lapse in vivo micro-CT reveals differential patterns of peri-implant bone changes after subclinical bacterial infection in a rat model
Source: Sci Rep. 2020 Dec 1;10:20901. doi: 10.1038/s41598-020-77770-z (PMC7708479; doi:10.1038/s41598-020-77770-z)
Supplement: Supplementary file 1 — Supplementary information. [file 41598_2020_77770_MOESM1_ESM.docx]

Longitudinal time-lapse *in vivo* micro-CT reveals differential patterns of peri-implant bone changes after subclinical bacterial infection in a rat model

Vincent A. Stadelmann^a,b^ vincent.stadelmann@kws.ch

Keith Thompson^a^ keith.thompson@aofoundation.org

Stephan Zeiter^a^ stephan.zeiter@aofoundation.org

Karin Camenisch^a^ karin.camenisch@balgrist.ch

Ursula Styger^a^ ursi.styger@aofoundation.org

Sheila Patrick^c^ S.Patrick@qub.ac.uk

Andrew McDowell^d^ a.mcdowell@ulster.ac.uk

Dirk Nehrbass^a^ dirk.nehrbass@aofoundation.org

R. Geoff Richards^a^ geoff.richards@aofoundation.org

T. Fintan Moriarty^a^* fintan.moriarty@aofoundation.org

^a^ AO Research Institute Davos, Clavadelerstrasse 8, CH-7270 Davos Platz, Switzerland

^b^ Schulthess Klinik, Department of Research and Development, Lengghalde 2, CH-8008 Zurich, Switzerland

^c^ The Wellcome-Wolfson Institute for Experimental Medicine, Queen’s University Belfast, Lisburn Rd, Belfast BT9 7AE, United Kingdom

^d^ Nutrition Innovation Centre for Food and Health (NICHE), School of Biomedical Sciences, Ulster University, Cromore Road, Coleraine, BT52 1SA, United Kingdom

# * Corresponding author

T. F. Moriarty, PhD

AO Research Institute Davos

Clavadelerstrasse 8, CH-7270 Davos Platz, Switzerland. Phone: +41 81 414 2397; E-mail: fintan.moriarty@aofoundation.org

*Short running title:* time-lapse *in vivo* microCT and subclinical infection

*Declarations of interest*: none

Supplementary Figure 1. Bacteriological outcome of infection. After euthanasia at day 28 post-implantation, rats were dissected and separate CFU counts were performed on the implant, overlying soft tissue and the bone (data from each location presented here). Data shown is from 3/3 animals per group, with the exception of *C. acnes* (pooled) with 14 animals in total. Historical *S. aureus* results are also shown for comparison. Horizontal lines indicate median values per group. Due to the logarithmic nature of the y axis, culture-negative samples were assigned an arbitrary value of 1.

Supplementary Figure 2: Quantitative analysis of longitudinal microCT assessment. In the main document, bone remodeling is presented as a single parameter, BF/BR. This data shows the corresponding bone formation and bone resorption for Figure 4, 7 and 9. In the upper row, bone formation follows a similar trend for sterile and *C. acnes* of rapid increase until 6 to 9 days to a peak at 0.3-0.4 mm3/day. For *S. aureus* and *S. epidermidis*, Bone Formation plateaus at 0.2 and 0.3 respectively already by day 3. Bone resorption is notably higher in *S. aureus* and *S. epidermidis* compared to the other groups. In the middle row, there is no clear difference between the patterns of BF or BR with respect to the sub-species of *C. acnes*. Finally (bottom row), a difference between culture positive and culture negative curves is only found in resorption.
